# Atypical squamous cells, cannot exclude high grade squamous intraepithelial (ASC-H) in HIV-positive women

**DOI:** 10.4103/1742-6413.64376

**Published:** 2010-06-12

**Authors:** Pam Michelow*, Ingrid Hartman, Doreen Schulze, Stella Lamla-Hillie, Sophie Williams, Simon Levin, Cynthia Firnhaber

**Affiliations:** Department of Anatomical Pathology, Cytopathology Unit, University of the Witwatersrand and National Health Laboratory Service, Johannesburg, South Africa; Department of Medicine, University of Witwatersrand, Clinical HIV Research Unit, Johannesburg, South Africa; Department of Obstetrics and Gynecology, University of Witwatersrand, Johannesburg, South Africa

**Keywords:** ASC-H, HSIL, HIV, low-resource community

## Abstract

**Objective:**

South Africa has very high rates of both HIV infection and cervical pathology. The management of ASC-H is colposcopy and directed biopsy, but with so many women diagnosed with HSIL and a dearth of colposcopy centres in South Africa, women with cytologic diagnosis of ASC-H may not be prioritized for colposcopy. The aim of this study was to determine if HIV-positive women with a cytologic diagnosis of ASC-H should undergo immediate colposcopy or whether colposcopy can be delayed, within the context of an underfunded health care setting with so many competing health needs.

**Materials and Methods:**

A computer database search was performed from the archives of an NGO-administered clinic that offers comprehensive HIV care. All women with a cytologic diagnosis of ASC-H on cervical smears from September 2005 until August 2009 were identified. Histologic follow up was sought in all patients.

**Results:**

A total of 2111 cervical smears were performed and 41 diagnosed as ASC-H (1.94%). No histologic follow up data was available in 15 cases. Follow up histologic results were as follows: three negative (11.5%), five koilocytosis and/ or CIN1 (19.2%), ten CIN2 (38.5%) and eight CIN3 (30.8%). There were no cases of invasive carcinoma on follow up.

**Conclusion:**

The current appropriate management of HIV-positive women in low-resource settings with a diagnosis of ASC-H on cervical smear is colposcopy, despite the costs involved. In the future and if cost-effective in developing nations, use of novel markers may help select which HIV-positive women can be managed conservatively and which ones referred for more active treatment. More research in this regard is warranted.

## INTRODUCTION

The Bethesda 2001 classification divides atypical squamous cells into two categories viz. atypical squamous cells of undetermined significance (ASC-US) and atypical squamous cells, cannot exclude a high grade squamous intraepithelial lesion (ASC-H). ASCH denotes cellular changes that are suspicious but not diagnostic of a high grade squamous intraepithelial lesion (HSIL). This diagnostic category includes a mixture of true HSIL and its mimics.[1,2]

South Africa has one of the worst HIV/AIDS epidemics worldwide.[3] South Africa also has high rates of cervical cancer and its precursor lesions.[4] HIV-positive women have higher prevalence of human papillomavirus (HPV) and infection with multiple HPV types, higher rates of squamous intraepithelial lesions that may be multifocal and recur after treatment and present at an earlier age with invasive squamous carcinoma, often at a later stage that their HIV-negative counterparts.[5‐10]

South Africa, like many other low-resource countries, has a dearth of both colposcopes and personnel trained in colposcopy. There are long waiting lists for women requiring colposcopy, in addition to transport issues to the relatively few centres offering colposcopy. There have also been a number of studies that reported a significant number of patients (23–28%) with a cytologic diagnosis of ASC-H required multiple colposcopies for definitive diagnosis of HSIL, further adding to the cost.[11] In an ideal situation, all women with ASC-H should be referred for colposcopy. However, given the scarcity of resources in South Africa and high prevalence of HSIL on cervical smear, women with a cytologic diagnosis of ASC-H may not be prioritized for colposcopy. In the literature, the rate of concurrent and subsequent HSIL on follow up of ASC-H is reported to be 29–75% and it is recommended that women diagnosed with ASC-H undergo colposcopy. However, this means that 25–71% of women with a cytologic diagnosis of ASCH do not have HSIL on follow up, with colposcopy being unnecessary and expensive in these women. Recently, there have been some studies investigating the use of HPV testing in women with an ASC-H diagnosis in an attempt to reduce the number of referrals to colposcopy. Unfortunately, HPV testing is currently unaffordable within the South African public health sector.[11‐18]

This study was undertaken to determine if HIV-positive women with a cytologic diagnosis of ASC-H should undergo immediate colposcopy or whether colposcopy can be delayed, in an underfunded health care setting, with so many competing health needs. As far as we are aware, this is the first study to examine ASC-H in an HIV setting.

## MATERIALS AND METHODS

Women for this study were part of the South African Cervical Cancer Cohort (SACCC). This is a five- year prospective, observational study of HIV-positive women examining the course of cervical cytologic abnormalities. The SACCC is based in the Themba Lethu HIV care and treatment clinic in a secondary government hospital in Johannesburg, South Africa that offers comprehensive HIV care (voluntary counseling and testing, adherence training, antiretroviral treatment, support services), TB/HIV/STI integrated services, referrals to and from other health facilities. HIV-positive women are treated according to HIV South African guidelines i.e., highly active retroviral therapy (HAART) is initiated at WHO stage 4 or CD4 count <200 cells/mm^3^.

The study population consisted of 2,111 women who underwent conventional cervical cytology between September 2005 and August 2009. Smears were taken with a cervical brush and fixed using a spray fixative. Liquid based cytology is not available within the South African public health sector. HPV testing is not routinely available and was done on six smears at the time of initial Pap smear. Smears were reported according to the 2001 Bethesda System.[1] The smears were screened by a cytotechnologist or cyto-technician and final report was by a cytopathologist. Ethics approval to conduct the study was obtained from the University of the Witwatersrand Human Research Ethics Committee (Medical). Women were offered a cervical smear after an educational session presented in English and Zulu and patient consent was obtained before enrolling participants in the study. For quality assurance purposes, a random sample of 10% of conventional cytology slides were sent to the University of North Carolina for blinded double-reading on two occasions which yielded a high rate of concordance (81–85%). In accordance with South African public health sector practice, women were referred for colposcopy for a cytologic diagnosis of HSIL or ASC-H. Treatment with LEEP was done if histology results found on colposcopy were CIN 2 and more advanced.

## RESULTS

**Figure 1:**
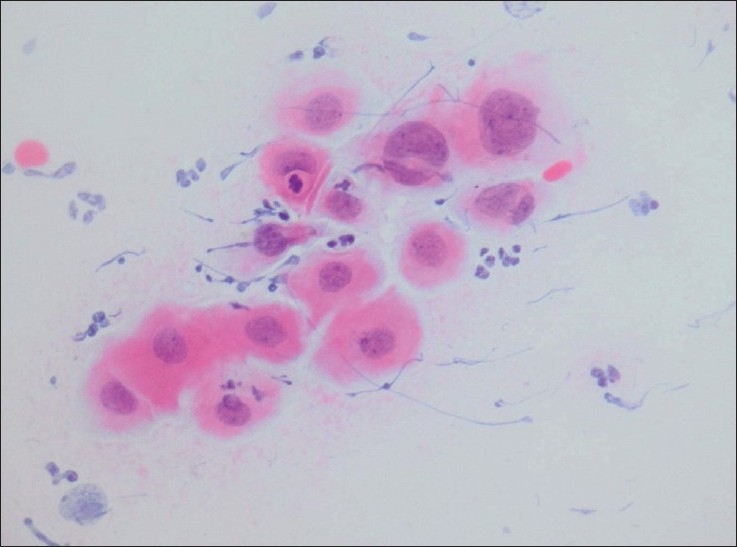
Metaplastic cells with an increased nuclear-cytoplasmic ratio and moderately hyperchromatic chromatin that on biopsy showed CIN1 (Conventional smear, Papanicolaou stain × 600).

**Figure 2:**
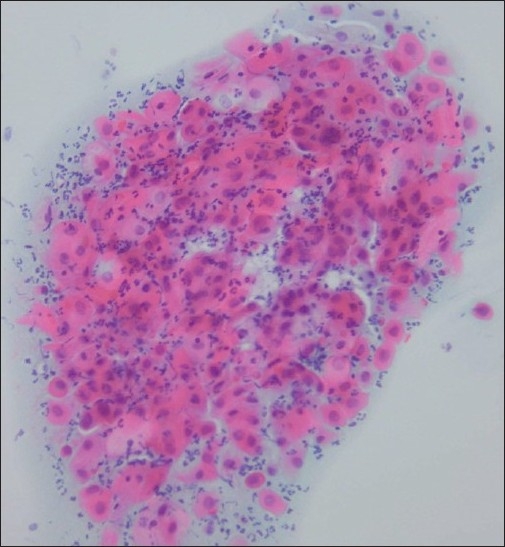
A single cluster of atypical cells reported as ASC-H that on biopsy showed koilocytosis only (Conventional smear, Papanicolaou smear × 400)

**Figure 3:**
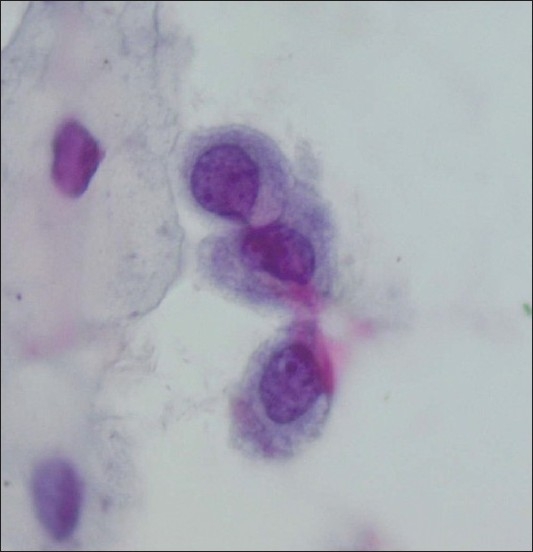
ASCH/immature squamous metaplasia that on biopsy showed immature squamous metaplasia with no evidence of HSIL (Conventional smear, Papanicolaou stain × 600)

**Figure 4:**
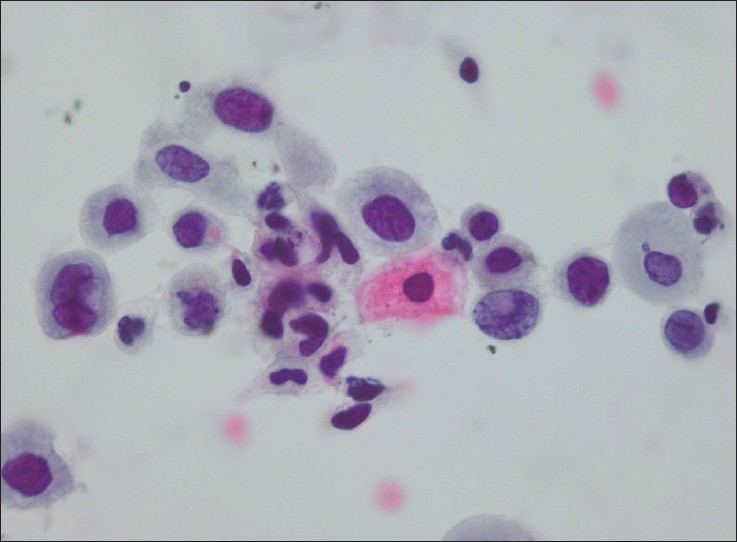
Metaplastic cells with irregular and hyperchromatic nuclei that on histologic follow-up showed CIN2 (Conventional smear, Papanicolaou stain × 600)

There is an extremely high burden of cervical disease in this study population which has been described previously.[6] There were 2,111 Pap smears, 232 (11%) inadequate and 1122 reported as abnormal (53.2%). There were 422 (19.9% of total and 37.6% of abnormal smears) cases of ASCUS, 305 LSIL (14.4% of total smears and 27.2% of abnormal smears), 41 ASC-H (1.9% of total smears and 3.6% of abnormal smears), 345 HSIL (16.3% of total smears and 30.7% of abnormal smears), three invasive squamous carcinomas (0.14% of total smears and 0.27% of abnormal smears) and six atypical glandular cells (0.28% of total smears and 0.53% of abnormal smears). There was no follow up in 15 patients with a cytologic diagnosis of ASCH despite tracking efforts at the clinic. The age range for ASCH in all 41 women was 26 to 59 with an average age of 40 years. The average CD4 count in all patients with ASC-H was 339/mm^3^ with a range from 7 to 1,006/mm^3^. The average CD4 count in women with an HSIL diagnosis in the same population of 2111 women was 243/mm^3^, a significant difference compared to the CD4 counts in ASC-H (*P* = 0.01). On histologic follow- up of 26 women with a cytologic diagnosis of ASC-H, there were three women with negative histology, five showed koilocytosis and/or CIN1, ten CIN2 and eight CIN3 [Table 1 and Figures 1‐4]. There were no cases of invasive carcinoma. Six out of 41 women with ASC-H had HPV testing performed at time of initial Pap smear using Roche Linear Array HPV genotyping test (Roche Molecular Systems Inc., California, USA). These results are summarized in Table 2.

**Table 1:** Results of histology

*Age*	*Negative histology*	*Koilocytosis and/or CIN1*	*CIN2*	*CIN3*	*Total*
<29	0	1	0	1	2
30–39	2	2	4	4	12
40–49	1	2	6	2	11
50–59	0	0	0	1	1

**Table 2:** CD4 counts, histologic diagnosis and HPV types in six women with a cytologic diagnosis of ASC-H

*Age (years)*	*CD4 count/mm^3^*	*Histologic diagnosis*	*HPV types*
35	307	CIN2	16,45,58,82
32	65	CIN2	31,53,61
33	69	Not available	11,18,69
47	121	CIN3	18,53
37	497	CIN2	16
48	82	CIN1	62,73,81,82

## DISCUSSION

South Africa has one of the highest HIV/AIDS rates in the world. The South African annual antenatal HIV seroprevalence surveillance survey is an anonymous, cross-sectional survey targeting 15–49 year old pregnant women attending antenatal clinics within the public health sector. In 2008, 33,927 pregnant women from 1,457 sentinel sites participated. The national overall HIV seroprevalence in this survey was 29.3%.[3] South Africa contains less than 1% of the world's population but 17% of its HIV/AIDS patients (approximately 5.5 million HIV-positive people).[19]

Cervical cancer, and its precursor lesions, is due to infection with the human papilloma virus (HPV). Studies have shown that women co-infected with both HIV and HPV have higher prevalence of cervical abnormalities. These cervical lesions may be multifocal and have a higher tendency to recur after treatment than in HIV-negative women. In addition, HIV-positive women present with invasive cervical cancer, on average, 10–15 years earlier. While the majority of low grade squamous intraepithelial lesions regress spontaneously in HIV-negative women, HIV-positive women have lower rates of regression and more tendency to progress. Several studies from Southern Africa show a diversity of HPV types in HIV-positive women, in addition to harbouring more than one subtype.[5‐10] Thus, it is essential that HIV-positive women undergo cervical screening, a challenge in many low-resource communities. Moodley *et al*,[5] state that if rigorous cervical screening is not undertaken, the benefits of antiretroviral therapy may be diminished by an excess risk in cervical cancer.

The 2001 Bethesda System[1] separates the category of atypical squamous cells into atypical squamous cells of undetermined significance (ASC-US) and atypical squamous cells, cannot exclude a high grade squamous intraepithelial lesion (ASC-H). ASC-H is used when cells are suggestive but not diagnostic of a high grade squamous intraepithelial lesion. ASC-H may be diagnosed when metaplastic cells occur singly or in small fragments with an increased nuclear-cytoplasmic ratio, somewhat irregular nuclear membranes and mild nuclear hyperchromasia. Alternatively, ASC-H may present as thick tissue fragments consisting of crowded cells that are difficult to visualize. However, cytologic features of ASCH are subject to poor interobserver agreement that probably accounts for the differing incidence of HSIL following a diagnosis of ASCH.[12]

ASCH is an infrequent cytologic diagnosis with a reported incidence of 0.13–0.3% of all gynaecologic cytology specimens.[12,18] In current study, ASCH was diagnosed in 41 out of 2111 smears (1.9%), perhaps overcall on part of this laboratory. In the same patient population, HSIL formed 16.3% of total cases. Thus rates of both ASC-H and HSIL are high and while elevated rates of HSIL are well-described in HIV-positive women, the rates of ASC-H in an HIV-positive population have not been previously determined. There appears to be a higher risk of squamous intraepithelial lesion following a diagnosis of ASC-US in HIV-positive women, especially in women with low CD4 counts compared to HIV-negative women.[20] According to Solomon *et al*,[2] ASC-H should not form more than 10% of the total number of atypical squamous cells. In this study, ASC-H makes up 8.85% while ASC-US 91.15% of the total number of cervical smears reported as atypical squamous cells. This study was based on conventional cytology and many in the literature utilize liquid based cytology which may account for the higher rates in this study in addition to the effect of immunosuppression on HPV-related cervical disease. According to Sherman *et al*,[21] a diagnosis of ASC-H may reflect inadequate sampling of an HSIL lesion, small HSIL lesions or early development of HSIL. Moktar *et al*,[13] recognise that a cytologic diagnosis of ASCH, which on further investigation proves negative, may be due to misinterpretation of metaplastic squamous cells and reactive cell changes.

Younger patients with ASCH have a higher incidence of an underlying HSIL than postmenopausal women. This may be due to the difficulties of taking an adequate smear in a postmenopausal women or interpretative changes in an atrophic smear e.g. degenerative and inflammatory changes, bare nuclei and pseudoparakeratosis.[22,23]

In this study, follow up of these HIV-positive women with a cytologic diagnosis of ASC-H revealed three women with negative histology, five with koilocytosis and/or CIN1, ten with CIN2 and eight with CIN3 i.e. 69.2% of women had CIN2 or worse. Review of cytology of these cervical smears revealed, in the vast majority of cases, a paucity of abnormal squamous cells which may account for the diagnosis of ASC-H rather than HSIL. The occasional atypical cell tended to lie singly rather than in syncytial arrangements and appeared metaplastic in many instances. Occasional smears showed atypical bare nuclei. The nuclear-cytoplasmic ratios were high but the nuclear borders showed mild to moderate irregularities and the chromatin varied from bland to hyperchromatic and granular. Many of these smears were obscured by an inflammatory infiltrate. Review of smears that were diagnosed as negative or CIN1 again revealed a paucity of 'atypical' cells in a background of inflammation. The average CD4 count in patients with a negative histologic diagnosis, koilocytosis and/or CIN1, CIN2 and CIN3 was 276, 250.3, 437.8 and 496.6/mm^3^ respectively. These results appear contrary to expectations but the numbers are very small and not statistically significant.

The use of HPV testing to reduce the number of women, with a cytologic diagnosis of ASC-H, referred for colpscopy has been suggested. Srodon *et al*,[16] identified HSIL in a significantly greater proportion of patients who had HPV-positive ASC-H compared with HPV-negative ASC-H while Liman *et al*,[23] determined that a negative high risk- HPV test is an excellent predictor of the absence of HSIL in ASC-H cervical smears. Chivukula and Shidham[24] found that, in liquid based smears, ASC-H showed a spectrum of cytomorphologic patterns and the combination of cells resembling dysplastic cells together with high-risk HPV positivity could be interpreted as HSIL. However, the usefulness of HPV testing in HIV-positive women may be reduced. Previous HPV typing on 148 patients from this HIV-positive clinic population[6] demonstrated 83% of women had one or more oncogenic HPV types including 67% in women with negative cytology while the prevalence of oncogenic HPV types in women with a cytologic diagnosis of ASCUS or LSIL was 94%. Furthermore, when stratified by CD4 count, there was a significant risk of oncogenic HPV types is patients with a CD4 count less than 200 cells/mm^3^, irrespective of the cytologic findings. A study from Cape Town[5] assessing HPV prevalence in cervical smears from 109 HIV-positive women, found 69.7% of all women harbouring HPV. In addition, a study by Ng'andwe in Zambia[24] showed a strong association between positive HIV status and the prevalence of high-risk HPV types. A study by Baay *et al*. in Zimbabwe[25] found that certain HPV types (HPV types 11, 39, 43, 51, and 59) occurred more frequently in HIV-positive women suggesting that HIV co-infection may have an impact on HPV genotype distribution. Even if HPV testing was affordable in low-resource countries, the high prevalence of oncogenic HPV types in the smears of HIV-positive women probably precludes the use of HPV testing in this clinical setting. It is possible that tests such as p16^INK4A^, a tumor suppressor gene or Pro-ExC^®^ that detects aberrant S-phase induction may prove more useful in this population.[26‐28] These tests appear to be markers of disease, rather than infection and may help identify those women requiring referral to colposcopy and those who may be safely managed in a more conservative manner. This is in contrast to HPV testing that is an indicator of infection, the majority of which will clear spontaneously. This is an exciting new area in cytology but, like many new technologies, cost constraints may limit the application of these novel markers in low-resource countries.

A very recent study in Thailand investigated the feasibility of 'see and treat' in the management of ASC-H and concluded that selective use of 'see and treat' in women with ASC-H smears who have high grade lesions on colposcopy is possible.[29] Further studies in HIV-positive women are warranted.

In conclusion, the current appropriate management of HIV-positive women in low-resource settings with a diagnosis of ASC-H on cervical smear is colposcopy. In the future, novel markers may establish which patients with ASC-H may be managed conservatively and which should be referred for colposcopy. However, the cost effectiveness of these innovative markers compared to colposcopy will need to be carefully established for use in poorer nations.

## COMPETING INTEREST STATEMENT BY ALL AUTHORS

No competing interest to declare by any of the authors.

## AUTHORSHIP STATEMENT BY ALL AUTHORS

Each author acknowledges that this final version was read and approved. All authors of this article declare that we qualify for authorship as defined by ICMJE http://www.icmje.org/#author. Each author has participated sufficiently in the work and take public responsibility for appropriate portions of the content of this article.

## ETHICS STATEMENT BY ALL AUTHORS

Ethics approval to conduct the study was obtained from the University of the Witwatersrand Human Research Ethics Committee (Medical)

## EDITORIAL / PEER-REVIEW STATEMENT

To ensure integrity and highest quality of CytoJournal publications, the review process of this manuscript was conducted under a double blind model(authors are blinded for reviewers and reviewers are blinded for authors)through automatic online system.
